# Additive Manufacturing of Alumina-Based Ceramic Structures by Vat Photopolymerization: A Review of Strategies for Improving Shaping Accuracy and Properties

**DOI:** 10.3390/ma18112445

**Published:** 2025-05-23

**Authors:** Jia-Jun Zhao, Yun-Zhuo Zhang, Jia-Hao Li, Zi-Heng Wang, Wei-Jian Miao, Fan-Bin Wu, Shu-Qi Wang, Jia-Hu Ouyang

**Affiliations:** School of Materials Science and Engineering, Harbin Institute of Technology, Harbin 150001, China; 24s009048@stu.hit.edu.cn (J.-J.Z.); 21b909010@stu.hit.edu.cn (Y.-Z.Z.); 24s109246@stu.hit.edu.cn (J.-H.L.); 23s009051@stu.hit.edu.cn (Z.-H.W.); 23s009061@stu.hit.edu.cn (W.-J.M.); 23s109209@stu.hit.edu.cn (F.-B.W.)

**Keywords:** vat photopolymerization, Al_2_O_3_, debinding, sintering, additive manufacturing, stereolithography, digital light processing

## Abstract

Alumina is a polycrystalline oxide ceramic with different structures. Currently, α-alumina with a hexagonal close-packed stacking structure is mainly used for a variety of industrial applications. Alumina-based ceramics and composites have been widely used in various fields due to their excellent hardness, strength, creep resistance and good biocompatibility. However, it is difficult for Al_2_O_3_ ceramic components based on traditional preparation methods to meet the increasing industrial requirements, especially for applications such as precise multi-walled complex structures. Al_2_O_3_ ceramic additive manufacturing by vat photopolymerization 3D printing stands out owing to its ability to produce complex structures and tailored shaping accuracy/properties. The vat photopolymerization 3D printing of Al_2_O_3_ ceramics requires multiple steps including slurry preparation, photopolymerization shaping, debinding and sintering. Therefore, many efforts mainly focus on the strategies of optimizing the ceramic slurry formulation and the debinding/sintering process. This paper provides a scoping review to present optimization strategies for the above aspects of vat photopolymerization 3D printing, which creates a strong reference for researchers to improve the accuracy and properties of alumina parts. Finally, this review also states the main applications of Al_2_O_3_ ceramic components based on vat photopolymerization, and highlights the opportunities and challenges associated with this technology in the future. It is beneficial to understanding the future trends and policy directions of advanced manufacturing industry.

## 1. Introduction

Additive manufacturing technology is a good solution to achieve the production of precision workpieces with a geometrically complex structure [1,2], which realizes the solid freeform fabrication of various ceramics. In recent years, ceramic additive manufacturing has developed rapidly [3,4]. Compared with traditional molding methods, it has higher efficiency and shaping accuracy, less raw material waste, and most importantly, it makes it possible to achieve some complex ceramic structures which would have otherwise been difficult in the past [5,6,7]. Multiple additive manufacturing technologies have been used for ceramic material molding [8], such as selective laser sintering (SLS), selective laser melting (SLM) [9], fused deposition modeling (FDM) [10], 3D gel casting (3DGP) [11,12], direct ink writing (DIW) [13,14], binder injection (BJT) [15], indirect 3D printing technology [16], and vat photopolymerization (VPP including stereolithography (SLA), digital light processing (DLP), liquid crystal display (LCD), and two-photon polymerization (TPP))

Additionally, 4D printing introduces the “time dimension” that considers the interaction between materials and environment over time. Four-dimensional printing has also grown rapidly in recent years [17]. These methods have expanded the design space of precision and complex structures, and can also be combined with other methods to achieve higher performance requirements [18].

Among these methods, VPP is highly favored due to its advantages of rapid printing speed, low consumption, and high accuracy [19]. The main principle of VPP is that, under the action of ultraviolet light, the photoinitiator decomposes into active groups to trigger the polymerization of photosensitive resins, thus curing the parts. At present, VPP has been widely used in advanced structural ceramics such as Al_2_O_3_ [20], AlN [21], ZrO_2_ [22,23], SiC [24], Si_3_N_4_ [25,26,27], SiO_2_ [28], etc.

The additive manufacturing of complex ceramic components through vat photopolymerization (VPP) represents a compelling example of the interaction between different types of artifacts [29]. These artifacts include both fabrication objects (such as the VPP process itself) and conceptual tools, such as natural language, which mediates the design and technical understanding. In light of emerging notions of complexity, the VPP process can be interpreted as a form of “complexity compression,” in which the sophistication of materials, formulations, and operational strategies is condensed into functional structures that are both accessible and adaptable, thereby enabling new forms of resilience in advanced sociotechnical systems [30].

Alumina ceramics exhibit different crystal structures under different conditions [31]. The main crystal structures are α-Al_2_O_3_, β-Al_2_O_3_ and γ-Al_2_O_3_. α-Al_2_O_3_ belongs to the tripartite crystal system, in which O^2−^ shows close-rowed hexagonal stacking, and Al^3+^ fills in the octahedral voids. This close-packed lattice energy is larger, and has the most stable crystal structure and the most stable chemical properties. β-Al_2_O_3_ belongs to the hexagonal crystal system, which is a kind of alkali-containing aluminate. β-Al_2_O_3_ crystals show a layered structure, which contains Na^+^, and this kind of sparse interlayer structure is conducive to the free movement of Na^+^ and thus produces relaxation polarization, which makes β-Al_2_O_3_ ionic conductivity. γ-Al_2_O_3_ belongs to the tetragonal crystal system, and its crystal structure generally resembles the cubic close-packed structure, whilst O^2−^ anions occupy the apex of the cube and Al^3+^ cations are regularly filled in the voids. γ-Al_2_O_3_ has this kind of loose structure, so that it obtains a high specific surface area and improves the surface energy of powder. Therefore, the chemical activity is very high, and γ-Al_2_O_3_ is most widely used in the field of catalysis. α-Al_2_O_3_ is usually used in VPP progress due to its stable chemical properties, and every occurrence of Al_2_O_3_ in this paper is α-Al_2_O_3_ if not otherwise specified.

Alumina ceramics and composites have a wide range of applications in precision machinery [32], ceramic filters [33,34,35], ceramic substrates [36], biomedical implants [37], ceramic cores [38], chemical catalysis supports and many other structural/functional integration devices due to their excellent strength, hardness, creep resistance and superior physical performance. There are many traditional molding methods for alumina ceramics, which often involve preparing ceramic powders, shaping, and then sintering them using various molds. The most common processes include slip casting, injection molding, gel casting, direct coagulation casting and filtration slurry injection [39]. However, due to high hardness and brittleness of alumina ceramics, traditional forming methods are prone to cracking and struggle to produce some structurally complex workpieces. Since the digital revolution, the demands for structurally complex workpieces, instruments, and equipment in the industry and commerce have been increasing, and traditional forming methods have become inadequate, which definitely limit the practical applications of Al_2_O_3_ ceramics and composites [40]. For example, ceramic crowns, ceramic cores, or gradient functional structures cannot be molded or personalized with a high precision and a shape complexity using traditional molding methods.

**Figure 1:**
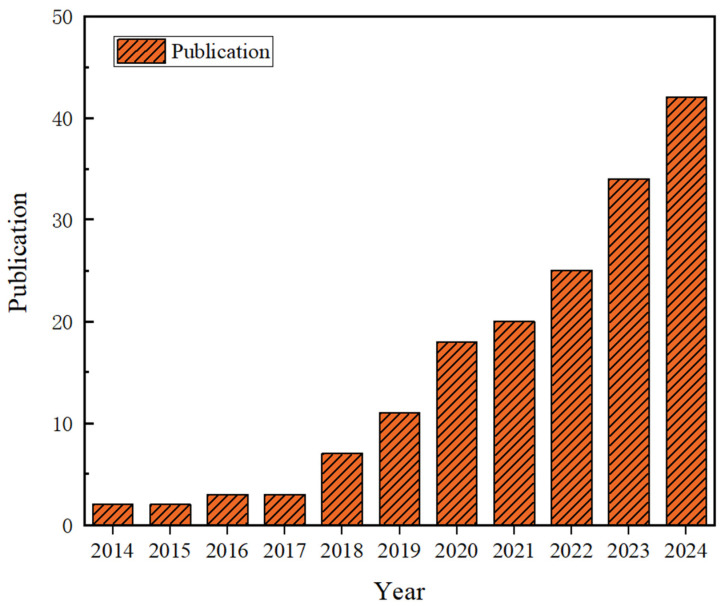
The ten-year trend of SCI indexed publications related to VPP-printed Al_2_O_3_ ceramics (data source from the web of science).

Al_2_O_3_ ceramics and composites based on vat photopolymerization have a broad application prospect due to its better refractive index match as well as its excellent mechanical and physical properties. In the future, Al_2_O_3_ ceramics will play an increasingly important role in various industrial fields [41,42]. Figure 1 shows the ten-year trend of SCI indexed publications related to VPP-printed Al_2_O_3_ ceramics. As seen in Figure 1, the publications related to VPP-printed Al_2_O_3_-based ceramics have been increasing year by year, which suggests that VPP is one of the important directions for the rapid prototyping of high-performance Al_2_O_3_ ceramics and composites in the future.

**Figure 2:**
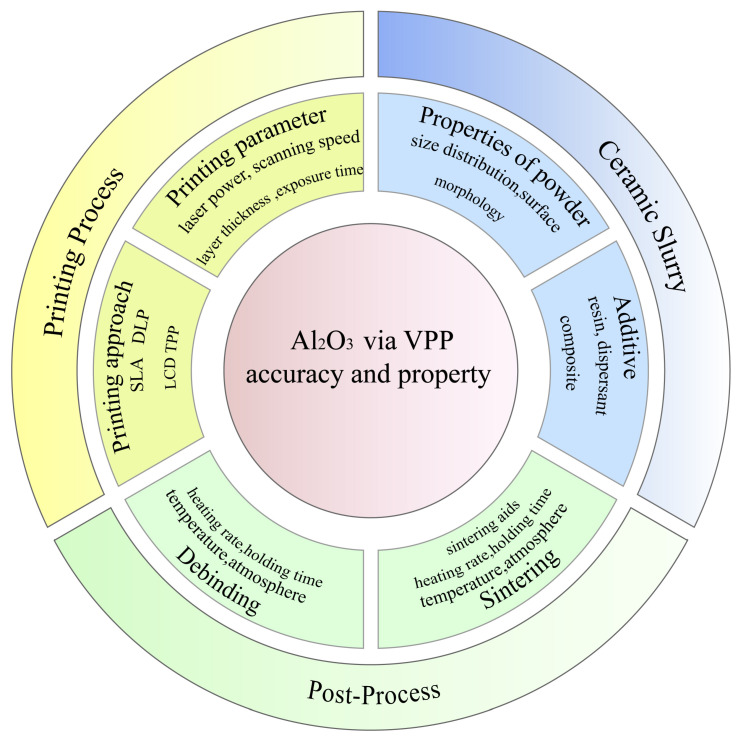
The factors affecting the VPP of Al_2_O_3_ ceramics. Specific influencing factors are as follows: the preparation of the ceramics slurry, printing process, and post-processing. Alumina ceramic slurry is a foundation of the printing process. Properties of powder, resin, and dispersant influence the rheological properties of the slurry. The shaping accuracy is greatly dependent upon printing approaches and parameters. The process design of the post-processing (debinding and sintering) has a great influence on the final properties.

Through VPP, not only dense Al_2_O_3_ components but also porous Al_2_O_3_ ceramic parts with complex structures such as skeletons, honeycombs, and microlattices can be produced [43,44], which greatly broadens the applications of Al_2_O_3_ ceramics [45]. The aim of this review is to introduce the state of the art of Al_2_O_3_ ceramics by VPP, including the principle of VPP and various aspects from Al_2_O_3_ ceramic slurry improvement to debinding and sintering optimization, and to explain the effects of these factors on the shaping accuracy and properties. Finally, the applications and the challenges of Al_2_O_3_ components based on VPP are pointed out. Figure 2 shows the factors affecting the VPP of Al_2_O_3_ ceramics. Every step affects the accuracy and properties of Al_2_O_3_ via VPP, including the slurry preparation, printing and post-processing treatments. This paper provides a scoping review to present optimization strategies for the vat photopolymerization 3D printing Al_2_O_3_ ceramics. It aims to render a strong reference for researchers to improve the accuracy and properties of Al_2_O_3_ parts and point out the opportunities and challenges within this field.

## 2. Principle of 3D Printing Ceramics Based on Vat Photopolymerization

VPP has been studied in depth before and is widely used in various fields, and has many advantages such as high efficiency, flexible design, convenient operation, high energy usage, and environmentally friendly protection. Slurry preparation is the first step in the VPP-3D printing of Al_2_O_3_ parts. After preparing the slurry, the desired part is modeled using a computer software, and the model is sliced and cured for printing layer by layer. Photosensitive resin monomer in the UV irradiation and photoinitiator under the joint action of polymerization to cure the ceramic slurry [46]. The process involves photoinitiation, chain growth, chain transfer, and chain termination [47]. After molding, the raw parts are debinded and sintered to obtain the final parts. This paper discusses optimization strategies in the preparation of alumina ceramic parts according to the preparation sequences of printing, debinding, and sintering.

**Figure 3:**
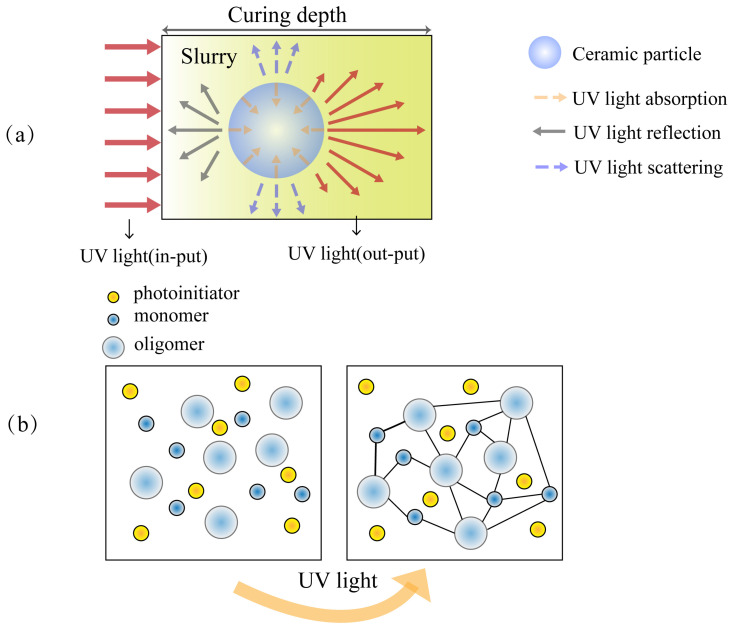
Interaction between the ceramic particles and light and the principle of VPP: (**a**) the interaction between ceramic particles in a ceramic slurry; and (**b**) the principle of VPP.

UV light is refracted, scattered and absorbed within the ceramic particles [48]. Figure 3 shows the interaction between ceramic particles and UV light and the principle of the VPP process. The refractive index match between ceramic particles and resin determines the performance of light curing. The refractive index of the photosensitive resin is around 1.5, while the refractive index of Al_2_O_3_ is around 1.75, which is not a big difference. Certainly, this feature is a major advantage of producing Al_2_O_3_ components with complex structures using the VPP method.

Photoinitiators are the core of photoinitiation. Photoinitiators decompose in the presence of UV light at the appropriate wavelengths to produce free radicals or cations. Photosensitive resins often have polymerizable functional groups such as unsaturated double bonds. When the reactive group attacks the polymerizable functional group, a chain reaction is initiated and polymerization begins [49]. Commonly used photoinitiators include BPB, Irgacure 819 and TPO, etc. A new photoinitiator called TMO (2,4,6-Trimethylbenzoyl) was applied to the Al_2_O_3_ ceramic slurry by Yao et al. [50]. The incorporation of TMO improves the shaping accuracy associated with the curing process, which exhibits excellent potential to cure the Al_2_O_3_ ceramic slurry. Commonly used photosensitive resins and dispersants will be discussed in detail in the Section 3.3.

The viscosity of the ceramic slurry is a crucial factor influencing vat photopolymerization [19]. The overall rheology cannot affect the kinetics of photopolymerization, which is only determined by the viscosity of the inter-particle phase, based on the kinetic analysis of ceramic photopolymerization process, as reported by Badev et al. [51]. The refractive ratio between the ceramic particles and the organic matrix affects the yield and rate of the photopolymerization reaction, whereby the higher the refractive ratio, the weaker the light penetration ability, the less thoroughly the vat photopolymerization reaction proceeds, and the smaller the depth of curing.

The Beer–Lambert law reflects the relationship between the optical absorption and the intensity of exposure [52]:

*C*_d_ = *D*_p_ ln (*E*/*E*_c_)

where *C*_d_ is the curing depth of the ceramic slurry, *E* is the exposure intensity, *E*_c_ is the exposure intensity that exactly cures the ceramic slurry, and *D*_p_ is the decay length.

Curing depth is an important indicator of the success of the photopolymerization reaction, as light curing is a layer-by-layer printing process, the curing depth should be guaranteed to at least 10–35% of the thickness of each layer, in order to avoid the phenomenon of printing delamination [53].

**Figure 4:**
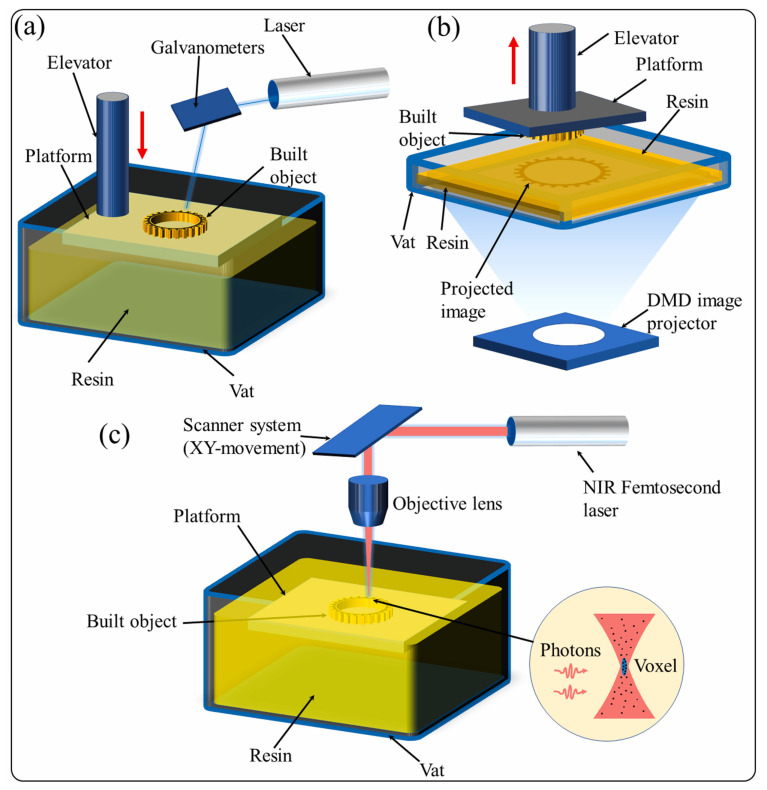
Schematic diagrams of different VPP technologies: (**a**) SLA; (**b**) DLP; and (**c**) TPP [64], copyright 2021, with permission from Elsevier.

Specifically, ceramic 3D printing derives more techniques and methods on the principle of photopolymerization [54]. This review concentrates on SLA, DLP, LCD (liquid crystal display), and TPP. Table 1 summarizes the advantages and disadvantages of different VPP methods. Figure 4 shows the schematic diagrams of different VPP technologies.

SLA (stereo lithography apparatus) is an early development of VPP 3D printing technology. The specific operation principle is a UV laser source under the control of a computer that executes the point-by-point irradiation of the liquid slurry underneath to achieve photopolymerization. The specific operation steps are, first of all, to fill the liquid tank with resin and then direct the laser beam onto the photopolymer surface, from the point to the line, from the line to the surface of a layer of solidification, and once a layer of solidification has been achieved, the construction platform will be reduced by a layer of thickness along the Z axis, before activating the squeegee to scrape to level the layer and the next layer of the print [53,55]. This process is repeated for each layer in turn until the 3D parts are printed. Due to the point-by-point scanning, it is more accurate and often achieves precise dimensional control and a glossy surface, but it is not efficient due to its slower curing rate, and Meana et al. [56] found that the total time to print the Al_2_O_3_ reference spherical structures using SLA was quite long.

**Table 1:** Advantages and disadvantages of different VPP methods.

Method	Resolution	Advantage	Disadvantage	Refs.
SLA	25–50 μm	High quality, high precision, and smooth surface	Slow speed, limited print size, and sensitivity to environmental conditions	[57]
DLP	10–50 μm	High precision, high detail representation, and fast speed	Limited print size and high resin cost	[58,59,60]
LCD	40–50 μm	Low cost and fast speed	Limited LCD screen lifespan and limited print size	[61]
TPP	Micro/sub-micron	Ultra-high precision, no support structures needed	Slow speed, high equipment cost complex technical complexity	[62,63]

DLP (digital light processing) is a computerized process that slices and dices the 3D model to be realized and solidifies it layer by layer using a series of light sources. The specific operation is to fill the resin tank with resin, and then expose the whole model to light using the optical digital projector to solidify the model layer by layer. For specific operations, there are two types of models including both the top–down and bottom–up configurations. The bottom–up paradigm requires less fresh resin in the resin tank and allows for the printing of small objects in containers using less resin. It can be adapted for more viscous resins; however, separating the polymerized layer from the resin tank substrate is a critical step in the printing process [65]. In contrast, in the top–down paradigm, the amount of low-viscosity resin required is higher. This helps to adjust the position of the build head in the case of a resin tank with a resin layer on top. An advantage of the top–down configuration is that there is no adhesion problem between the layer and the resin tank substrate because the resin polymerizes on the free surface and comes into contact with air. However, polymerization may be inhibited in the projection region due to the contact of ambient oxygen with the resin surface [66,67].

DMD (digital micromirror device) is a series of light sources, consisting of thousands of microscopes pointing in a single direction across the projected area of the cross-section, which can result in the total curing of a given cross-section, which improves the efficiency of the light polymerization [68].

LCD (liquid crystal display) is similar to the DLP method, but utilizes a liquid crystal LCD screen light source for the DMD. This method is also a layer-by-layer curing of the material with higher efficiency, and reduces the cost of 3D printing. However, the uniformity of the LCD light source is not good, and curing at some of the edges of the region is not sufficient, which affects the printing accuracy.

TPP (two-photon polymerization) is a high-precision additive manufacturing technology, mainly used for printing micro- and nano-structures [62]. The specific principle is to make the material absorb two photons at the same time to reach the excited state by the femtosecond laser, and at the laser focus, the photosensitive resin undergoes the polymerization reaction [63]. Two-photon absorption is a nonlinear process that occurs only when the light intensity at the laser focus is high enough, thus enabling high-resolution printing. The two-photon polymerization device builds three-dimensional complex structures layer by layer by moving the laser focus or the printing platform [64].

## 3. Optimization of Al_2_O_3_ Ceramic Slurry

Alumina-based ceramic slurries are carefully prepared prior to VPP printing. In fact, in recent years, some researchers tried to use sol–gel precursors instead of slurry for printing. For example, Moshkovitz-Douvdevany et al. [69] used Al_2_Cl_3_ precursor for VPP printing, and then a heat treatment process was used to obtain Al_2_O_3_ ceramics from photopolymerizable sol–gel compositions. This avoids the high requirements of the ceramic slurry properties for 3D printing, and may be one of the future directions. However, most of the current studies still focus on the improvement of the ceramic slurry performance, so this review will focus on the improvement strategies of Al_2_O_3_-based ceramic slurries.

### 3.1. Composition of Al_2_O_3_ Ceramic Slurry

The performance of photopolymerized Al_2_O_3_ ceramic slurry is a main factor affecting the quality of 3D printing, and the photopolymerized Al_2_O_3_ ceramic slurry mainly includes photosensitive resin, photoinitiator, dispersant, sintering aids, and Al_2_O_3_ ceramic powder. In some studies, in order to obtain porous Al_2_O_3_ ceramics such as ceramic films, catalysts, and biomimetic structures, pore-forming agents are often added to the slurry. For example, Chen et al. [44] added polyamide 12 (PA12) as a porous agent into the ceramic slurry and obtained high-resolution porous Al_2_O_3_ ceramics; Nohut et al. [70] fabricated VPP-printed alumina-based ceramics with spatially resolved porosity and mechanical properties comparable to those of conventional processes using PMMA (methyl methacrylate) as a porous agent.

VPP-based 3D printing requires high rheological properties of ceramic slurry, including low viscosity and long dispersion stability. Ceramic particles should ideally be uniformly dispersed in the slurry. Therefore, the choice and content of the dispersants are also quite important, which will be analyzed in the Section 3.3. The better ceramic slurry for VPP-printing needs low viscosity to ensure the flow during the printing process and not to settle in in a short time. At the same time, a lower viscosity of the slurry exhibits less resistance to flow, which can improve the shaping accuracy of VPP-printed parts. Unstable ceramic slurry that settles in a short period of time can lead to inhomogeneous properties and compositions in the as-printed parts.

Another important factor to examine is the solid content in the slurry, since no components other than ceramic powders in the slurry play a role in realizing the properties of components, so VPP-printed slurries require the highest solid content possible, while the subsequent debinding and sintering processes also need to form components with a high solid content [71,72]. Liu et al. [73] found that the higher the solid content of the slurry, the lower the shrinkage after sintering and the higher the density. In addition, temperature and ball milling speed are also important aspects to increase the solid content of the slurry. Increasing the temperature can significantly increase the solubility and reactivity [74]. However, when the solid content is too high, the ceramic particles in the slurry will scatter and absorb the incident light, which will affect the photopolymerization process, so it is important to try to increase the solid content of the slurry to ensure that the slurry has sufficient photopolymerization ability.

The selection of the solid content must also consider its effect on viscosity. In order to balance the relationship between viscosity and solid content, researchers have constructed a number of models. One of the more important models is the Krieger–Doherty model, a hard-sphere model. It provides the relationship between viscosity and solid content in the form of an equation and has a good experimental fit [75]:

η_r_ = η_s_/η_0_ = (1 − φ/φ_m_)^−*B*φm^

where η_r_ is the relative viscosity, η_s_ is the suspension viscosity, η_0_ is the medium viscosity, φ is the volumetric percent solids, φ_m_ is the maximum volumetric solids content (maximum filling), and *B* is the Einstein coefficient (also known as the intrinsic viscosity).

Therefore, improving Al_2_O_3_-based ceramic slurry has always been the focus of Al_2_O_3_ photopolymerization research. In this review, the effects of three aspects including powder properties, resin and dispersant, and composite ceramic slurry on Al_2_O_3_ ceramic slurry are specifically discussed.

### 3.2. Physical and Chemical Properties of Al_2_O_3_ Powders

As a major component of Al_2_O_3_-based ceramic slurry, the effect of Al_2_O_3_ powder properties on the slurry has received wide attention. The particle size distribution is an important characteristic of Al_2_O_3_ ceramic powders. Generally speaking, the larger the particle size of the powder, the better the slurry fluidity; while the smaller the particle size, the more obvious the agglomeration phenomenon is, which reduces the fluidity of the slurry. However, a large particle size easily leads to the insufficient stability of slurry settlement. Xu et al. [76] investigated the effect of the particle size of Al_2_O_3_ ceramic powders on the rheological properties of slurries. The selection of Al_2_O_3_ powders with a medium particle size (4 μm) resulted in the better integrated rheological properties with a viscosity of 1.62 Pa·s at 30 s^−1^. Although the use of Al_2_O_3_ ceramic powder with a larger particle size (8 μm) significantly reduced the viscosity of the suspension (1.02 Pa·s at 30 s^−1^) and increased the solid content of the slurry, the increase in particle size led to a rapid decrease in the stability of Al_2_O_3_ ceramic slurry. On the contrary, the use of alumina powder with a smaller particle size (2 μm) led to a remarkable increase in the viscosity of the slurry. Yang et al. [77] investigated the effect of nanoscale Al_2_O_3_ ceramic powders on the rheological properties of ceramic slurries. The nanoscale Al_2_O_3_ ceramic powders improve the stability of the slurry, and as the content of nanoscale alumina powders is relatively lower, these particles are uniformly dispersed in the slurry system. As the alumina content increases, the agglomeration of larger particles appears in the resin system, which leads to a significant increase in the viscosity of the slurry. The surface of the as-printed samples becomes rougher and rougher as the content of the nanoscale alumina increases. The particle size distribution also has a large effect on the interaction between the ceramic particles and UV light. It mainly affects the scattering process. As the particle size increases from the nano-scale to the micro-scale, the scattering mechanism changes from Rayleigh scattering to geometrical optics, and the scattering intensity of single particles becomes stronger, which reduce the curing depth. Therefore, an improvement in the rheological properties by changing the particle size distribution should be considered along with its light curing ability.

The morphology of Al_2_O_3_ powders also has an effect on slurry properties. Yang et al. [78] investigated the effect of Al_2_O_3_ powder morphology on the rheological properties of slurries. Al_2_O_3_ powders with lamellar and spherical structures, respectively, were used for individual slurry formulation and DLP printing. The ball milling process breaks up some of spherical Al_2_O_3_ powders, resulting in a decrease in fluidity. The relative density of the DLP-printed Al_2_O_3_ ceramics from spherical powders is 91.1% and the flexural strength is 92.1 MPa. In contrast, the laminar Al_2_O_3_ powders produce a directional effect during the flow process and have a lower viscosity. At the same time, this directionality makes the lamellar powders more tightly bound. The relative density of the DLP-printed Al_2_O_3_ ceramics from lamellar powders is 93.2%, and the flexural strength is 165.5 MPa. Camargo et al. [75] found that, the smaller the surface area of spherical Al_2_O_3_ powders, the lower the viscosity of the formulated slurry compared to irregularly shaped Al_2_O_3_ powders.

The surface modification of Al_2_O_3_ powder particles is also a direction to regulate the rheological properties of the slurry. The surface modification of alumina powders mainly serves to improve the dispersion of the slurry, and thus reduce the viscosity. Zhang et al. [79] studied the surface modification of Al_2_O_3_ powder particles, and added a surface modifier of Silane A174 into the slurry, whilst the modified Al_2_O_3_ particles had a better wettability with the hydrophobic photosensitive resins, and at the same time, dispersed KOS110 was used to form steric hinderance on the surface of Al_2_O_3_ particles to achieve effective dispersion. Based on steric repulsion stability, the final Al_2_O_3_ slurry has a solid content of 65 vol.% and a viscosity of 20.0 Pa·s at a shear rate of 30 s^−1^. Yun et al. [39] improved the dispersion of Al_2_O_3_ ceramic particles in the photopolymer solution using the method of encapsulating a silane coupling agent (VTES) on the surface of the powders for SLA 3D printing.

Overall, the characteristics of Al_2_O_3_ powders represent an important factor influencing the performance of slurry for VPP-based printing. The selection of suitable powder particle size distribution, morphology, and surface properties is quite beneficial for improving the rheological properties and printability of Al_2_O_3_ slurry.

### 3.3. Resins and Dispersants

The selection of resins and their contents is an important direction for improving the Al_2_O_3_ slurry [75]. Photosensitive resins generally have polymerizable functional groups such as unsaturated double bonds to ensure that they can undergo chain reactions. Besides, the resin monomer is required to have a low viscosity and a good compatibility with Al_2_O_3_ ceramic powder so as to ensure the uniform dispersion in the solvent and improve the rheological properties of the slurry [80]. Currently, different types of acrylic and acrylate monomers are commonly used. The most commonly used acrylate monomer is the HDDA monomer.

The number of reactive functional groups is a crucial factor in polymerization. Multi-functional monomers promote cross-linking during polymerization and improve the strength and hardness of printed parts compared to mono-functional monomers. However, a higher number of functional groups also cause an increase in viscosity, and monofunctional monomers are also known as reactive dispersants due to their low viscosity properties.

Xing et al. [81] used silane couplers of KH550, KH560, and stearic acid (SA) as surfactants for Al_2_O_3_ particles, and the low-viscosity acrylic monomers of ACMO, HDDA, and NPG2PODA as reactive diluents for Di-TMPTA-based pre-mixed resins, and succeeded in preparing well-dispersed Al_2_O_3_ slurry with a viscosity of 25 Pa·s at a shear rate of 30 s^−1^ and a relatively high solid content of 44.2 vol%. Oezkan et al. [47] enhanced the curing depth of the body to 1.2 mm by increasing the functionality and molecular weight of the monomers in the blends, and optimum viscosity and mechanical properties were obtained when the difunctional monomers were mixed with trifunctional (meth)acrylates. The ultimate tensile strength reaches 55.41 MPa, while the viscosity of the slurry is only 19.33 mPa·s (at 200 r/min).

The affinity of the liquid medium to Al_2_O_3_ particles is the basis for the homogenization of the suspension. Generally, oxide particles such as Al_2_O_3_ have hydroxyl groups on their surface, which makes their powders hydrophilic. Most ceramic slurries are formulated in non-aqueous formulations due to a greater depth of light curing compared to aqueous solutions. However, the hydrophilic nature of ceramic particle surfaces reduces the dispersibility of powders in nonpolar media. One way to solve the affinity problem is to make the hydrophilic surface of the oxides compatible with the hydrophobic medium by means of a dispersant [80]. On the other hand, ceramic slurries generally have a high solid content, which leads to a sharp increase in the viscosity of the slurry due to van der Waals force attraction between the ceramic particles, which is very unfavorable for subsequent printing, and therefore also requires a viscosity modulation by dispersants [82].

**Figure 5:**
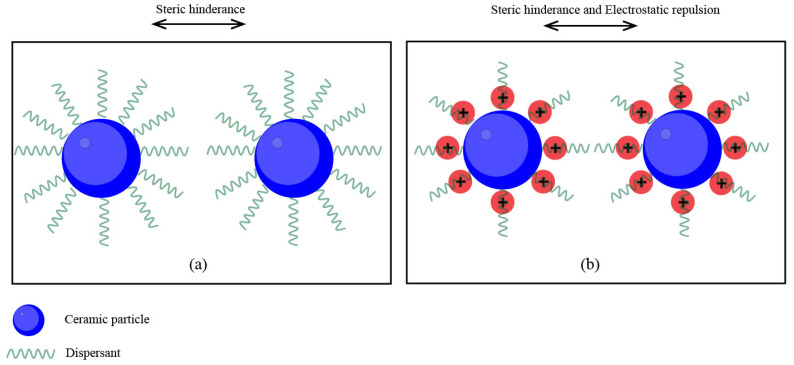
Two mechanisms of dispersants: (**a**) steric hinderance; and (**b**) steric hinderance and electrostatic repulsion (“+” stands for electric charge) [80], copyright 2024, with permission from Elsevier.

Therefore, the use of dispersant is a key consideration in the formulation process of Al_2_O_3_ ceramic slurries. Dispersants are generally organic substances with long chains. There are two main mechanisms by which dispersants act. The first one is electrostatic repulsion. If the surface of the particles is charged, a repulsive potential is generated, but this is related to the distance between the particles, and it is difficult to completely disperse the particles by electrostatic repulsion alone. The other mechanism is steric hinderance, which prevents the aggregation of two neighboring particles by adsorbing long organic molecules on the surface of particles, which generates a repulsive force when their polymer layers overlap. In this way, the dispersant enhances the stability of the slurry, and can better meet the requirements of photopolymerization [83,84]. Figure 5 shows the two mechanisms of dispersants.

Dispersants play a very important role in improving the solid content in ceramic slurry. Yu et al. [85] used the rheological dispersants of Uniqsperse-9450/9510/9012/93370 to regulate the rheological properties of Al_2_O_3_ slurry, and prepared a ceramic paste with up to 85 wt.% solid loading, which was successfully printed using the SLA method.

Morita et al. [86] investigated the 3D structure of the dense Al_2_O_3_ ceramics using SLA, and enhanced the light-curing performance of Al_2_O_3_ slurries using polyethyleneimine (PEI) and oleic acid (OA) partial complexes as reactive polymer dispersants. The slurries with a higher solid content (35 vol.%) and a lower OA content (15 mol.%) in the PEI-OA composition are more favorable for light curing at a lower resin percentage.

Alves et al. [87] investigated the effects of three different dispersants of Castament FS 10, Triton-X and BYK-111 on the rheological properties of Al_2_O_3_ slurry with different solid concentrations. The dispersion of BYK-111 was considered to be the most effective in reducing the viscosity of the suspension, producing optimized results when the solid concentration was increased to 5.0 wt.%. This is attributed to the fact that BYK-111 introduces more hydrophilic segments to attach to the Al_2_O_3_ particle surface, while the hydrophobic segments form a spatial barrier that prevents the proximity of other particles, thus effectively reducing the viscosity of the slurry. Table 2 summarizes the commonly used resins and types of dispersants in Al_2_O_3_ slurry.

It is also important to choose a suitable content of dispersants. Zhang [79] studied the effects of different contents of KOS110 on the viscosity of photosensitive Al_2_O_3_ slurry. The viscosity of Al_2_O_3_ slurry decreases as the increase in shear rate, which appears to be characteristic of shear thinning. The viscosity is lowest (3–3.6 Pa·s) when the content of KOS110 is 5 wt.% at the same shear rate, and the rheological properties of slurry are the best.

### 3.4. Al_2_O_3_-Based Composite Ceramic Slurry

Al_2_O_3_-based composite ceramic slurries have received more and more attention in order to obtain better-performing Al_2_O_3_ ceramics via photopolymerized 3D printing to meet the needs of more application scenarios. However, it is still challenging to incorporate other ceramic powders or dissimilar materials into the Al_2_O_3_ ceramic slurry, because the determination of the relevant parameters of the composite slurry requires more exploration and experimentation, otherwise it is difficult to achieve the viscosity and stability required for photopolymerization. Table 3 summarizes the rheological properties of the Al_2_O_3_ composite slurry.

The rheological properties of the Al_2_O_3_ composite slurry are the first thing to consider. Aktas et al. [97] doped 5 wt.% TiO_2_ into Al_2_O_3_ powders and tested the rheological behavior of composite slurries with different solid loadings of 45, 50, and 55 wt.%, respectively, whilst the viscosity of ceramic slurries correspondingly increases to 380, 920, and 1640 mPa·s. The optimum solid content for the TiO_2_-doped Al_2_O_3_ composite slurry was finally determined to be 50 wt%. Due to its high solid content and low viscosity, this compositional formulation meets the criteria for DLP of TiO_2_-doped Al_2_O_3_ ceramics. Wu et al. [98] investigated the rheological behavior of Al_2_O_3_-ZrO_2_ composite slurry. The viscosity increases exponentially when the solid content in the Al_2_O_3_-ZrO_2_ composite slurry reaches 65 wt.% or more, and the lowest viscosity of only 0.2 Pa·s is achieved when the content of the dispersant PVP is 1.2 wt.%. Therefore, a composite slurry with a solid content of 65 wt.% and a PVP content of 1.2 wt.% is selected to successfully produce an Al_2_O_3_-ZrO_2_ ceramic body by SLA. Coppola et al. [99] mixed different ratios of ZrO_2_ and Al_2_O_3_ to prepare composite slurries, and successfully produced dense Al_2_O_3_-ZrO_2_ composite ceramics via DLP-based stereolithography followed by debinding and sintering. Nie et al. [102] combined the chemical precipitation coating process with the SLA to refine and homogenize the microstructure of 3D-printed Al_2_O_3_ ceramics. Y_2_O_3_ is coated on the surface of Al_2_O_3_ particles. The coated Al_2_O_3_ powder results in a more homogeneous and well-refined microstructure of 3D printed Al_2_O_3_ ceramics. The coating of Y_2_O_3_ on particles increases the curing depth of the body, without adversely changing the viscosity of the slurry as contrasted with uncoated Al_2_O_3._

Additionally, the influence of various composite components on the photopolymerization process is also a very important topic. Tanska et al. [95] added Mo/Ni into Al_2_O_3_ slurry, and found that the addition of metals strongly hinder the light curing owe to their high extinction coefficients.

Inserra et al. [100] added Ce-ZrO_2_ into Al_2_O_3_ ceramic slurry, and explored the relationship between the rheological behavior and the curing depth at different Ce-ZrO_2_/Al_2_O_3_ solid contents of 50–62.5 wt.% during DLP. The composite slurry exhibits a shear-thinning characteristic, and the curing depth decreases with an increasing solid content, which is due to the fact that Ce-doped zirconia in the composite slurry has a large obstruction effect on the ultraviolet light. From Table 3, it can be seen that the Ce-doped ZrO_2_/Al_2_O_3_ slurry exhibits a decrease in the curing depth as contrasted with unmodified ZrO_2_/Al_2_O_3_ slurry. Therefore, it is quite necessary to consider the solid content and the curing depth as a compromise in order to realize the smooth printing of Ce-doped ZrO_2_/Al_2_O_3_ composite slurries.

Gu et al. [101] incorporated graphene into Al_2_O_3_ ceramic slurry for top–down DLP 3D printing, and investigated the effect of graphene content on the UV absorption properties of Al_2_O_3_ slurry. Under UV irradiation at a wavelength of 405 nm, the reflectance of the cured Al_2_O_3_ body decreases with the increasing graphene content, which indicates that the incorporation of graphene could improve the UV light absorbance of Al_2_O_3_ slurry with high-solid-loading. The UV light absorbance increases with the increase in graphene content. Generally, in the UV range, the darker the powder color, the higher the UV absorbance. The graphene powder is uniformly dispersed in the slurry and partially adsorbed on the surface of Al_2_O_3_ particles, forming the absorption center of UV light, thus enhancing the UV absorbance of the slurry. However, a lesser graphene content would greatly increase the viscosity of the slurry (as shown in Table 3), so the contradiction between UV absorbance and rheological behavior should be carefully considered when choosing the content for the DLP 3D printing of high-performance and high-precision Al_2_O_3_ architectures.

Wang et al. [103] incorporated trace amounts of carbon fibers into Al_2_O_3_ ceramic slurry, and strictly controlled the content of carbon fibers (this is due to the fact that carbon fibers will reduce the laser penetration to affect the light curing) to evaluate the effect of carbon fibers on the properties of a ceramic body. The 3D-printed Al_2_O_3_ parts incorporated with carbon fibers by VPP exhibit different degrees of improvements in several performance parameters. Particularly, the samples incorporating 0.2 wt.% carbon fiber content showed the most significant toughness improvement (up to 0.8 MPa·m^1/2^).

In general, the Al_2_O_3_-based composite slurries need to fully consider the characteristics of the dopant component itself and the complicated impacts on the system, and sometimes, the dopant components can meet the target demands such as strength and fracture toughness, but may generate the negative impact on other photopolymerization properties of the slurries, such as viscosity and stability. It is necessary to carefully consider the types and contents of additives, or the use of other reagents to improve the performance of the composite slurries. In addition, Al_2_O_3_ whiskers or coatings are often added as a component to other light-curing ceramic slurry systems to enhance performance or adjust the refractive index [104,105].

## 4. Effect of VPP Process on Properties and Accuracy of 3D-Printed Al_2_O_3_ Ceramics

### 4.1. Structural Design and Printing Process Parameters

Before printing, the researcher needs to model and slice the structure of the printed parts. The design of the 3D-printed structure is an important part of VPP molding. The advantage of VPP 3D printing is the ability to build complex structures that are difficult to obtain by traditional methods. This requires the use of computer modeling. Modeling software, such as CAD, allows researchers to design and optimize a variety of topologies. The topology design not only enables its efficient use, such as structural weight-reducing and materials-saving, but also allows the performance to meet the requirements under extreme environments [106]. By artificially improving the structure, the stress distribution of components is optimized and multifunctional integration is achieved. This review introduces several optimization methods of topology design: (1) Porous structure design: Reduce material usage by enhancing porosity; (2) Lattice structure design [107]: Mimic lattice structure to achieve lightweight and high-strength requirements; (3) Biomimetic design: Design to take advantage of novel structures that exist in nature. In recent years, there has been an increasing number of studies on the construction of optimized topological Al_2_O_3_ structures via VPP.

**Figure 6:**
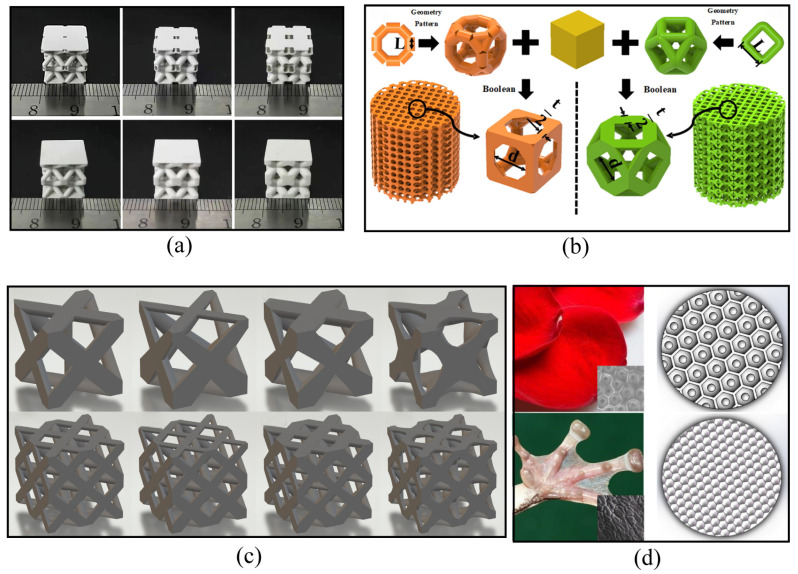
Some of the topological structures of VPP-printed Al_2_O_3_: (**a**) two Al_2_O_3_ ceramic lattice structures (hollow lattice structure and solid lattice structure) [90], copyright 2021, with permission from Elsevier; (**b**) two types of porous scaffolds models: octagonal and rhombic [108], copyright 2022, with permission from Elsevier; (**c**) the models of Al_2_O_3_ ceramics with functional gradient structures [93], copyright 2022, with permission from Elsevier; and (**d**) bionic petal structures and tree frog toe structures models for DLP-printed Al_2_O_3_ [109], copyright 2020, with permission from Elsevier.

For example, Zeng et al. [93] designed and printed alumina ceramics with functional gradient structures. These structures can achieve the maximum energy absorption of 2.72 × 10^5^ J/cm^3^. And, the compressive strength is 19.62 MPa. Lei et al. [108] designed and printed two types of porous scaffolds, octagonal and rhombic, by DLP method. The modulus of elasticity of different alumina-based lattice structures for bone implants ranges from 0.5 to 4 GPa, which meets the requirements of mechanical properties of bone trabeculae in the range of 0.1 to 4.5 GPa. Sun et al. [90] prepared two Al_2_O_3_ ceramic structures (hollow/solid lattice structure) via DLP method. The compressive strength of the solid block with a diameter of 2.0 mm and a porosity of 45% was 9.70 MPa. In contrast, the compressive strength of the hollow grating with a diameter of 2.0 mm and a porosity of 70% was 4.30 MPa. Chen et al. [109] designed different bionic petal structures and tree frog toe structures for DLP-printed Al_2_O_3_ ceramics. Figure 6 shows some of the topological structures of VPP-printed Al_2_O_3_.

The printing of the Al_2_O_3_ ceramic body is a critical step in vat photopolymerization molding, which depends not only on the rheological properties of the slurry and light curing ability, but also on the specific operation and parameters of light curing. Light is transmitted, scattered, and refracted through the ceramic particles, with the decreasing intensity perpendicular to the printed layer. If the printing parameters are not selected correctly, this can lead to both poor interlayer bonding and the cracking of the body, as well as excessive volume contraction and overcuring, resulting in a loss of dimensional accuracy [110,111,112].

The main printing parameters that need to be controlled in VPP are laser wavelength and power, scanning speed, layer thickness, and exposure time. Scanning speed and laser power have a large effect on the curing depth and width. The laser wavelength needs to be matched to the photoinitiator. Li et al. [113] found that ceramic prepared with higher curing depths showed the excellent surface quality of Ra < 1.6 μm and lower molding accuracy. The increase in the curing depth improves interlayer adhesion. However, excessive curing depth leads to a decrease in monomer conversion from 5.2% to 2.0% and an increase in internal stress. Additionally, increasing the curing depth exacerbates the pyrolysis process, and increases the tendency for crack formation. Decreasing the scanning speed and increasing the laser power are beneficial for enhancing the curing depth and width. The small scanning spacing of a laser beam may lead to the curling of the body due to excessive light curing in a small spacing; however, an excessively large spacing will affect the curing link and weaken the strength of the body. Therefore, it is important to choose a right scanning spacing.

The preset layer thickness is a key parameter. Too thin a layer is prone to excessive light curing and warpage, while too thick a layer may lead to insufficient curing, which affects the strength of the body (always between 25 and 200 μm). Li et al. [114] investigated the control of the crack propagation mode by means of finite element analysis. As the thickness of the printed layer increases, the layer structure gradually appears, the pores of the interlayer interface are gradually concentrated from uniform distribution to the interfacial pore line, and the crack extension changes from microcracks at the grain boundary to macroscopic cracks in the interlayer, which is a good reference for the selection of printing parameters.

Additionally, the choice of exposure time is also critical. With the increasing exposure time, the utilization of its light energy decreases. As the exposure time increases, the curing thickness increases, and the highest precision is achieved when the exposure time makes the ceramic particles have the smallest scattering effect on light [65]. The printing parameters should also take into account the print size issue, as it is a layer-by-layer curing, the light curing size is too large, and there is a high probability that the curing will be insufficient to affect the printing accuracy [115]. Negi et al. [116] found that shorter exposure times are more suitable for printing elaborate part geometries because scattering is more pronounced at longer exposure time; and at the beginning of the printing process, a long exposure time is required for curing and making the initial layer adhere to the build platform. After a few layers are printed, the exposure time can be reduced. However, when the exposure time is too short (<100 s), interlayer peeling can be observed.

In conclusion, the printing process parameters for Al_2_O_3_ ceramic parts mainly affect the printing accuracy and warpage degree of the body, and thus the performance of the final product. The influence of environmental factors such as humidity and temperature should also be considered during the actual printing process.

### 4.2. Influence of the Debinding Process for Al_2_O_3_ Ceramic Body via VPP

The printed Al_2_O_3_ ceramic body contains organic substances such as photosensitive resins that do not contribute to the final ceramics. Therefore, debinding and sintering are required to obtain the final product after light curing. Debinding and sintering are collectively referred to as post-processing treatments. Table 4 lists the effects of debinding and sintering processes on the properties of Al_2_O_3_ via VPP.

There are various debinding methods such as thermal debinding, solvent debinding, catalytic debinding, microwave debinding, supercritical debinding, and so on. In the case of Al_2_O_3_ ceramic body via VPP, thermal debinding and a combination of other methods with thermal debinding is generally used in an oxidizing or nonoxidizing atmosphere or under a partial vacuum. For example, Nurmi et al. [120,121] introduced supercritical carbon dioxide (scCO_2_) extraction in thermal debinding to create a flow path for the pyrolysis gases of the remaining binder polymer to exit the body. The supercritical carbon dioxide extraction at 140 bar and 40 °C extraction conditions achieved the highest average mass removal without causing any defects. This could significantly save the thermal debinding preconditioning time for photopolymerization, and improve the productivity of Al_2_O_3_ ceramic bodies. In the meantime, the extraction of scCO_2_ enhances the flexural strength and density of Al_2_O_3_ parts, and the overall accuracy is significantly improved. The relative density and flexural strength of the as-obtained Al_2_O_3_ ceramic are 97.2% and 320 MPa, respectively.

Thermal debinding is a process of removing organic matter by means of heat to obtain a pure ceramic body, which is a non-steady state transfer of heat into the body and the mass transport of the decomposition products out of the body. The debinding process is influenced by both chemical and physical factors, and can be divided into three stages. In the early stage of heating, the body still contains a considerable amount of resin, due to the volatilization of organic matter, the surface of the body gradually becomes rough; At the second stage, the majority of the resin in the body is removed, the formation of pores in the body greatly increases; At the third stage, all the resins are almost removed, the porosity of ceramic no longer change, and a more loose composition is created [122].

If the proportion of resin is high, the porosity produced in the loose ceramic body is too high after debinding, and may be difficult to bear their own weight and collapse; Secondly, the volatilization of organic matter will produce internal stress in the body during the debinding process. So, if the internal stress is too high, it may lead to the cracking of the body, which will seriously affect the properties of the finished product. The magnitude of the generated internal stress is highly related to the intensity of the debinding process, so controlling the rate of resin pyrolysis to inhibit cracking is still the main research direction. The debinding atmosphere, temperature, heating rate, and process all have a strong influence on the debinding process [123].

Temperature, heating rate, and holding time are the preferred factors to consider. The decomposition temperature and decomposition products are chemically determined according to the temperature range of the decomposition of the resin mixture in the ceramic slurry. The decomposition temperature of the resin can be determined experimentally by thermogravimetric analysis (TGA). From Table 4, the debinding temperature of the Al_2_O_3_ ceramic slurry for VPP is roughly between 600 °C and 1200 °C. The heating rate mainly controls the intensity of debinding, and excessively fast heating may trigger local overheating or a violent reaction to produce defects. The holding time controls the completion of debinding. The holding time should be chosen in consideration of the resin removal rate. Li et al. [124] investigated the effect of vacuum debinding on the microstructure and the properties of Al_2_O_3_ ceramics at different heating rates. As the heating rate increased, the inter-layer spacing increased. Pore diameter, shrinkage, flexural strength and hardness decrease with increasing heating rate. The grain size, phase composition, chemical bonding structure, bulk density, and open porosity of 3D printed Al_2_O_3_ ceramics varied with the heating rate. The optimum heating rate of 0.5 °C/min is selected to obtain a high flexural strength of 27.5 MPa.

The debinding atmosphere is an important subject. Air debinding can accelerate the oxidation of the resin and promote the debinding. But, the process is more violent and prone to cracks. Debinding in an inert atmosphere or vacuum avoids the violent and exothermic oxidation reaction, but is prone to produce residual carbon. In inert atmospheres such as N_2_ or Ar, resins undergo thermal degradation by chain scission to form smaller segments, or undergo depolymerization reactions to produce a high percentage of volatile monomers.

Liu et al. [91] designed a two-step debinding process in air and N_2_ atmospheres. The relative density of Al_2_O_3_ ceramics is significantly improved to 96% by the two-step degreasing process in different atmospheres followed by sintering. Chen et al. [125] found that the average grain size of alumina ceramics debinded under Ar was larger than that obtained by debinding directly in the air, the microstructure was dense without obvious pores and heterogeneous phases, and the as-sintered ceramics had a higher compressive strength. This indicates that the alumina ceramics obtained after debinding and sintering in Ar have a better performance. The highest relative density of alumina ceramics debinded in Ar is 96.72%, while the compressive strength is 761.7 MPa followed by final sintering. Li et al. [126] also investigated the optimum debinding temperature of Al_2_O_3_ ceramics under N_2_ atmosphere as 500 °C, and no evidence of residual carbon was observed above this temperature for debinding.

Wu et al. [127] investigated the effects of the particle gradation and debinding method on the debinding process of 3D-printed Al_2_O_3_ parts based on stereolithography. Micron-sized and nanosized Al_2_O_3_ powders were mixed in the ceramic slurry and exhibited the highest densities after debinding; however, the lowest densities were obtained with micron-sized Al_2_O_3_ powders alone, which was due to the lower surface energy of the micron-sized Al_2_O_3_ powders. The debinding in a vacuum environment resulted in higher densities of Al_2_O_3_ ceramic bodies than normal thermal debinding. The combination of the bimodal particle size distribution of the powders with a vacuum debinding process provides an effective method to improve the densification of Al_2_O_3_ via VPP.

Defects produced during debinding are one of the most important indicators of the success of the debinding process. The occurrence of nonuniform shrinkage, distortion and bubble formation may significantly influence the ability to control the shape and structural uniformity of the Al_2_O_3_ ceramic body. Nonuniform shrinkage occurs due to the Al_2_O_3_ particle rearrangement process to achieve a denser packing under the action of surface tension of the resin melt. However, a lower solid loading, higher resin content, and lower melt viscosity may lead to the distortion and warping of Al_2_O_3_ ceramic body. Bubble formation results from the decomposition of the resin as well as the incomplete elimination of residual products, which provides a possible source of failure or defect formation during thermal debinding. Investigating the debinding mechanisms and thus improving the resin system to minimize cracking is a feasible option. Kim et al. [128] proposed a mechanism to explain the formation of defects during the debinding process. The small molecules of uncured resin monomers decompose at low temperatures (<325 °C) to form gas removal, which may lead to the formation of defects. Such defects formed at low temperatures are very harmful to the body. They proposed the use of a non-reactive diluent, polypropylene glycol (PPG), as an additive in the curable resin to successfully prepare defect-free Al_2_O_3_ photopolymerized 3D-printed ceramics. PPG crosslinks uncured monomer small molecules, preventing them from decomposing into gases and escaping at low temperatures, and PPG preferentially decomposes into gas pores during debinding, which is conducive to the proximity and escape of the decomposed gases from small molecules without defects. Zhou et al. [129] used dibutyl phthalate (DBP) to optimize the composition of the resin formulation, and the DBP addition not only reduces the content of other binder components, but also promotes the formation of porous microstructures during the debinding process and further prevents the generation of distortion and cracking.

### 4.3. Influence of the Sintering Process for Al_2_O_3_ Ceramic Body via VPP

The debinded Al_2_O_3_ ceramic body needs to be finally sintered at high temperature to achieve the desired performance (including mechanical and other physical properties, etc.), which is undoubtedly key to achieving full densification. During the sintering process, the Al_2_O_3_ ceramic body becomes denser, the grain morphology evolves from rounded spherical to equiaxed, and the porosity disappears as the sintering temperature increases [130], which tends to undergo volume shrinkage after sintering due to the pores left by debinding. The best performance of Al_2_O_3_ ceramic components was reported at a sintering temperature of 1600 °C [131]. The VPP performs layer-by-layer molding, which in turn makes the anisotropic shrinkage of the body and is not conducive to the dimensional accuracy of the final molding. Therefore, the sintering process must be designed to ensure the demand of accuracy and properties.

The shrinkage properties of the final products can be optimized through improvements in the sintering process. In general, with the increasing sintering temperature and holding, the shrinkage of the Al_2_O_3_ parts increases. The higher the solid contents in the slurry, the lower the shrinkage of the final parts. Li et al. [132] optimized the sintering process of 3D-printed alumina ceramic cores by improving the number of sintering cycles. The use of a triple sintering method with the pore-forming agent make the shrinkage of ceramic parts in all three directions smaller and more uniform. The triple sintering method also significantly improves the final flexural strength of alumina ceramic cores up to 39.8 MPa. Li et al. [133] used a new method of atmosphere-controlled in situ oxidation of Al powder for the first time to achieve 3D-printed Al_2_O_3_ ceramic cores with near-zero shrinkage in the X direction. This method regulates the in situ oxidation reaction of Al powder by changing the atmosphere from Ar to air. The alumina ceramic cores are then prepared by liquid-phase sintering with the participation of atmospherically protected molten Al.

Tailoring the grain size is also one of the key concerns during sintering. As seen from Table 4, the ceramic parts with smaller grain sizes have better properties despite different applications and process parameters. Hofer et al. [134] used a radiation-assisted sintering (RAS) method to achieve the rapid sintering of Al_2_O_3_ ceramics at a high rate of temperature increase, which distinctly refines the grains in final sintered ceramics. Carloni et al. [135] used a two-step vacuum sintering method to increase the density. Compared to the single-step sintering method, two-step sintering refines the grain and improves transparency to 70%.

The sintering atmosphere mainly influences the formation of pores and cracks. Sintering in an inert atmosphere inhibits the generation of defects. Zhang et al. [136] sintered Al_2_O_3_ samples with different sintering cycles under four kinds of different atmospheres (air, Ar, and mixtures of Ar and vacuum). It is noteworthy that sintering under Ar and vacuum atmospheres did not generate large pores, cracks, or delamination, but resulted in a decrease in density.

Research on the sintering of 3D-printed Al_2_O_3_-based composites is also a major focus. The role of the composite component is mainly to introduce new phases and refine the grain, or to meet special performance requirements [137]. Table 5 shows the sintering process and the properties of 3D-printed alumina-based composites. By comparing Table 4 and Table 5, it can be noticed that the composites can be sintered at a lower sintering temperature and exhibit smaller grain sizes. Liu et al. [138] sintered VPP-printed Al_2_O_3_-ZrO_2_ composite bodies at 1500 °C for a holding time of 60 min. The actual density, hardness, and fracture toughness of the ceramics reached 3.75 g/cm^3^, 14.1 GPa, and 4.05 MPa·m^1/2^, respectively. However, when the temperature exceeds 1500 °C, abnormal grain growth occurs, which is detrimental to the improvement of properties.

In addition to changing the sintering process (e.g., temperature, time, atmosphere, etc.), many efforts have also been made to optimize sintering aids. Sintering aids serve a variety of purposes: (1) form a liquid phase themselves to promote diffusion and reduce the sintering temperature; (2) form a liquid phase with Al_2_O_3_ to improve the density; and (3) segregate at grain boundaries to prevent excessive grain growth.

Li et al. [142] studied the effects of different sintering aid types (TiO_2_, CaCO_3,_ and MgO) and the contents on the properties of Al_2_O_3_ ceramic parts. The flexural strength of the sintered Al_2_O_3_ ceramics increases dramatically from 19.2 MPa to 216.7 MPa while increasing the TiO_2_ content from 0.5 wt.% to 1.5 wt.%. TiO_2_ can form a solid solution with Al_2_O_3_, which also promotes solid-state sintering [143]. Due to the formation of elongated grains, the shrinkage of Al_2_O_3_ ceramics increases while increasing the content of CaCO_3_, which leads to the formation of large-sized residual pores. The addition of MgO helps reduce the anisotropy of both shrinkage and flexural strength of as-sintered ceramics owe to the formation of regularly shaped grains. Wei et al. [144] found that the addition of MgO-Y_2_O_3_ sintering aids significantly improves the densification process, and increases the flexural strength of Al_2_O_3_ ceramics. A flexural strength of 492 MPa and an apparent porosity of only 0.11% can be achieved with the addition of 0.5 wt.% MgO and 1 wt.% Y_2_O_3_.

The sintering temperature of Al_2_O_3_ ceramics is relatively high, which may affect the use of Al_2_O_3_ ceramic parts as various functional devices. Wang et al. [145] used 3.5 wt.% TiO_2_ and 3.5 wt.% CuO-Mg(OH)_2_-TiO_2_ as sintering aids in order to reduce the sintering temperature. The optimum temperature for sintering pure Al_2_O_3_ is 1600 °C and the flexural strength is 351 MPa. However, with the addition of 3.5 wt.% TiO_2_, the optimum sintering temperature is reduced to 1450 °C and the flexural strength is 328 MPa. In addition, with the addition of 3.5 wt.% CuO-Mg(OH)_2_-TiO_2_, the optimum sintering temperature is further reduced to 1250 °C, and the corresponding flexural strength is 301 MPa. Obviously, the effect of ternary additives on lowering the sintering temperature is better than that of a single additive. The effect of sintering additives on the properties of 3D-printed alumina ceramic parts is specifically shown in Table 6.

The defects produced during the sintering of Al_2_O_3_ ceramics are a key indicator of product performance. Nefedovaa et al. [147] compared the porosity after sintering of conventional slip casting and light-cured molded billets, and found that light-cured molded Al_2_O_3_ ceramics exhibited not only a lower porosity, but also a smaller pore size than those obtained by the conventional slip casting process. Guo et al. [148] conducted a study on vat photopolymerization-printed Al_2_O_3_ parts using two coating agents, E44 and E51, and found that the use of epoxy coating agents reduces the formation of defects, and improves the physical and mechanical properties of Al_2_O_3_ parts. At present, adding fibers/whiskers to the VPP-printed alumina material system to enhance its toughness and inhibit large crack extension is also one of the future directions [149].

## 5. The Properties, Applications, and Challenges of Al_2_O_3_ Ceramic via VPP

The advanced manufacturing industry has put forward higher requirements on the performance and accuracy of Al_2_O_3_-based composite parts via VPP, such as superior mechanical properties and geometric precision. Al_2_O_3_ ceramics produced by VPP have shown good application prospects in many fields such as aerospace, chemical catalysis, biomedical implants, friction and lubrication, and the electronics industry [150]. This review describes some key applications of VPP-based 3D-printed Al_2_O_3_ ceramics.

### 5.1. Ceramic Core

In the field of aviation, the cooling of turbine blades is a critical issue that directly affects the thermal efficiency of advanced engines. The cooling of turbine blades, in turn, relies on the internal serpentine complex structure, which is generally a silica-based ceramic core or an alumina-based ceramic core [151,152,153]. Although many efforts have been made on silica-based ceramic cores, the increasing operating temperatures greatly limits their applications in advanced engines. α-Al_2_O_3_, on the other hand, is increasingly exhibiting its superiority due to its stability at elevated temperatures. Ceramic cores have complex geometries, so many attempts have been performed to print Al_2_O_3_ ceramic cores via VPP. However, there still exists some technical difficulties associated with the VPP of Al_2_O_3_ ceramic cores that need to be overcome, such as the high linear shrinkage brought by the light-curing method and the difficulty of balancing the contradiction between porosity and high strength requirements [38].

**Figure 7:**
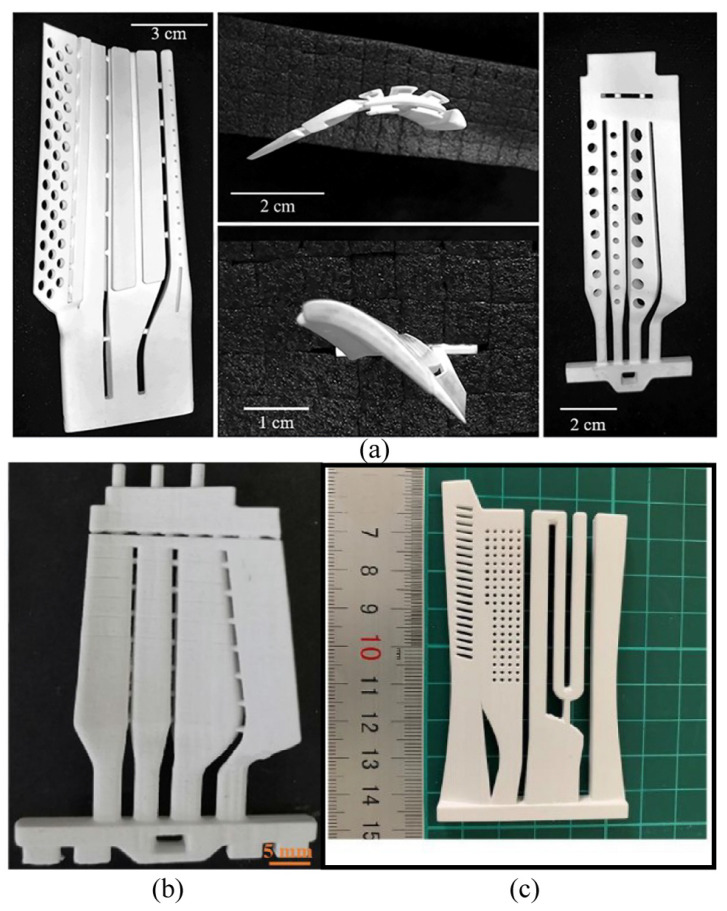
Some VPP-printed Al_2_O_3_ ceramic cores: (**a**) Al_2_O_3_ ceramic core printed by SLA [154], copyright 2022, with permission from Elsevier; (**b**) Al_2_O_3_ ceramic core printed by DLP [161], copyright 2022, with permission from Elsevier; (**c**) SLA-printed Al_2_O_3_ ceramic core with high porosity based on a powder gradation design [156], copyright 2022, with permission from Elsevier.

Various methods have been used to obtain high-porosity and high-strength Al_2_O_3_ ceramic cores with complex geometries. Table 7 listed different methods for modulating the properties of Al_2_O_3_ ceramic cores and product performance. Figure 7 shows some VPP-printed Al_2_O_3_ ceramic cores. In fact, since the core is a porous multilayered structure with relatively large dimensions, it is still a challenging issue to suppress the generation of defects/cracks during post-processing. The stress concentration due to the porous structure especially poses a risk for the reliability of alumina ceramic cores in practical applications.

**Table 7:** Methods for modulating the properties of Al_2_O_3_ ceramic cores and product performance.

Approach	Porosity (%)	Flexural Strength (MPa)	Shrinkage X Axis	Shrinkage Y Axis	Shrinkage Z Axis	Ref.
Bimodal particle size ceramic slurry	30.07	28.21	3.98%	-	-	[154]
Combining the VPP with sacrificial templating	36.93	75.4	7.91%	9.1%	12.51%	[155]
Powder gradation design	36.4	50.1	15.6%	-	-	[156]
Pore former and triple sintering	-	39.8	3%	4%	3%	[132]
Using SiO_2_ and Y_2_O_3_ as mineralizer	19.3	30.5	3.4%	3.1%	4.5%	[157]
Atmosphere-controlled in situ oxidation of aluminum powder	45.02	72.7	0.3%.	-	1.4%	[133]
Add CaO	50.4	24	23.5%	19.8%	16.5%	[140]
Vacuum sol impregnation	-	92.57	-	-	-	[158]
Y_3_Al_5_O_12_ (YAG)-enhanced alumina-based ceramic cores	40.8	16.1	3.8%	3.7%	4.8%	[159]
Buried combustion method	36.71	25	6.45%	7.67%	4.92%	[160]
Control sintering temperature(1400 °C)	35.1	20.3	-	-	-	[161]
In situ mullite-reinforce	40	25	5.2%	6.5%	6%	[162]

### 5.2. Chemical Catalysis

Applying the VPP method to produce Al_2_O_3_ ceramic catalysts is also a promising direction. Catalysts generally have to be guaranteed to have a large surface area to host active substances and therefore rely on high porosity. Based on VPP, such complex porous structures can be well realized; however, densification during high-temperature sintering tends to destroy this structure with a high specific surface area [163]. Suitable pore-forming agents or special processes are often selected to preserve the porous structure. Santoliquido et al. [164] designed a closed-wall Al_2_O_3_ lattice consisting of 2 mm rotating cubic cells by the SLA method for gas treatment in combustion engines. This structure achieves equivalent catalytic activity using only a smaller surface than conventional honeycomb-structured catalysts.

**Figure 8:**
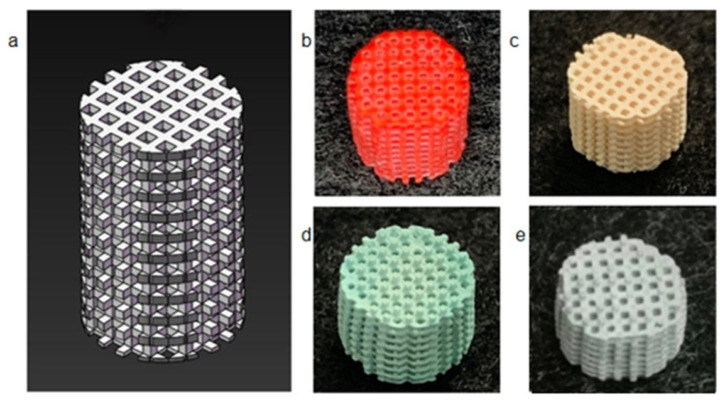
Key steps for the fabrication of Al_2_O_3_ catalysts [165]: (**a**) modeling in the calculator; (**b**) 3D printed structure; (**c**) carrier after sintering; (**d**) carrier after impregnation; and (**e**) structured Al_2_O_3_ catalyst, copyright 2021, with permission from American Chemical Society.

Wang et al. [165] proposed a VPP resin formulation for the fabrication of γ-Al_2_O_3_-structured catalyst carriers by DLP technology, using the ceramic slurry consisting of γ-Al_2_O_3_ and additives such as modified acrylates, which are debinded and sintered at lower temperatures. High-precision γ-Al_2_O_3_ catalyst carriers with complex structures were obtained, with a specific surface area of 163 m^2^/g, and exhibited an excellent catalytic performance in methanol hydrogen production. Figure 8 shows the key steps for Al_2_O_3_ catalyst fabrication.

Mastroianni et al. [166] used a new method to formulate a colloid with boron and then fully infiltrated the light-cured Al_2_O_3_ bodies in the colloid, and improved its mechanical properties by boron infiltration. Finally, it only needed to be calcined at 600 °C for 1 h to obtain Al_2_O_3_ ceramic catalysts with a high specific surface area, which proved to be catalytically active in the oxidative dehydrogenation of bioethanol. Kabakci et al. [167] successfully designed and fabricated Al_2_O_3_ structural supports for CO_2_ methanation reaction using SLA. Based on the twisted honeycomb geometry, the monolithic design was fabricated by SLA and sintered at 1300 °C. The proposed monomer design was able to reduce Ni loading while maintaining high methane yields, resulting in savings in material use and reactor production costs. The highest CO_2_ conversion of the twisted monomer with 0.5 mm hexagonal cells was 84% at 400 °C and 89% at 425 °C, respectively.

### 5.3. Friction, Wear and Lubrication

**Figure 9:**
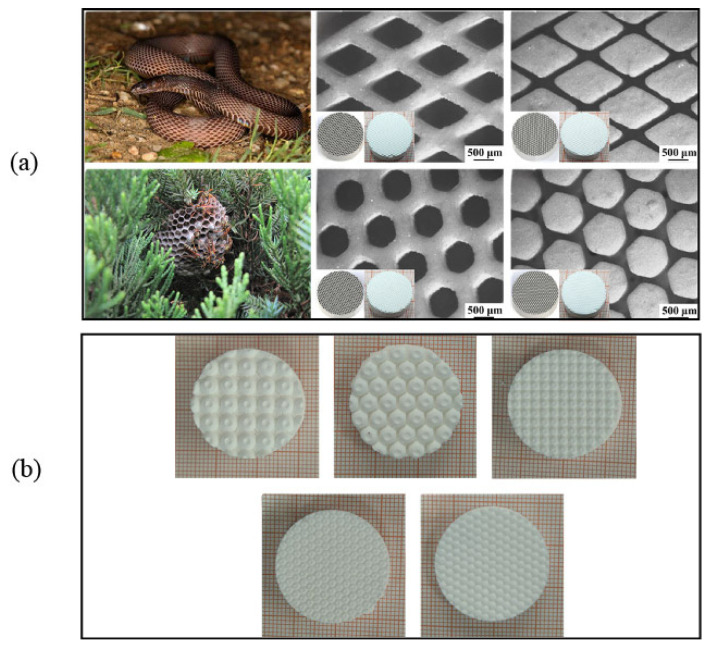
The structural design for tribological applications: (**a**) the serpentine skin-like structure and Al_2_O_3_ parts [170], copyright 2021, with permission from Elsevier; and (**b**) petal structures and tree frog toe structural Al_2_O_3_ parts [109], copyright 2020, with permission from Elsevier.

With the deepening of human/biomimetic tribology, the structural design of tribology is getting more and more attention. Many efforts have been made to improve the friction-reducing, wear-resistant and lubrication properties of components in sliding and rolling contacts by designing these complex structures. Al_2_O_3_ ceramics have inherently good wear and corrosion resistance; however, it is difficult to mold a complex structure of Al_2_O_3_ parts using traditional manufacturing methods. With the development of VPP, it has become possible to design the complex tribological structures of Al_2_O_3_ ceramics for specific application scenarios [168]. Figure 9 shows some structural design of tribology. The selection and design of an excellent structure is one of the difficulties in the tribological structure of VPP molding. In addition to borrowing potentially useful structures that exist in nature, researchers need to open up the boundaries of new structures based on guidelines such as topological structure design principles. These structures need to satisfy the need for structural/functional integration, which is a challenging topic that deserves attention in the future [169].

Zhao et al. [170] developed a serpentine skin-like bionic structure of Al_2_O_3_ ceramics by DLP, and investigated the corresponding synergistic lubrication properties with 2D-layered MoS_2_/*h*BN lubricants, as shown in Figure 9a. The honeycomb-like structured composites exhibit good tribological performances with a coefficient of friction of 0.31, which is 46.55% lower than unstructured Al_2_O_3_ samples. The serpentine skin-like composite structures exhibit an excellent friction coefficient of about 0.2 at 700 °C, which is nearly 88.51% lower than that of unstructured Al_2_O_3_.

Chen et al. [109] designed the different bionic petal structures and tree frog toe structures of DLP-printed Al_2_O_3_ ceramics, as shown in Figure 9b. The bionic petal structure has a better lubrication performance than the tree frog toe structure. Compared to the various bionic lubrication structures loaded with WS_2_, the bionic petallet structure with a hexagonal arrangement exhibited the lowest coefficient of friction of 0.411. The tree frog toe-end structure has a large coefficient of friction of 1.177, but shows good wear resistance.

All the bionic textured composites exhibit superior lubrication properties at different temperatures due to their larger contact area for restoring the lubricant and the formation of lubrication film as compared to unstructured Al_2_O_3_. This is a good indication for Al_2_O_3_ ceramics via VPP, which have a large potential for tribological structure design and fabrication.

### 5.4. Biomedical Applications

The biomedical field is filled with the need for personalized and customized structures. The development of reverse engineering has made it easier to obtain model structures that actually exist. Modeling costs for VPP-3D printing have been reduced. The extraction and printing of human body models is becoming a promising directions in the future, and VPP will become a powerful tool for personalized biomedical devices.

**Figure 10:**
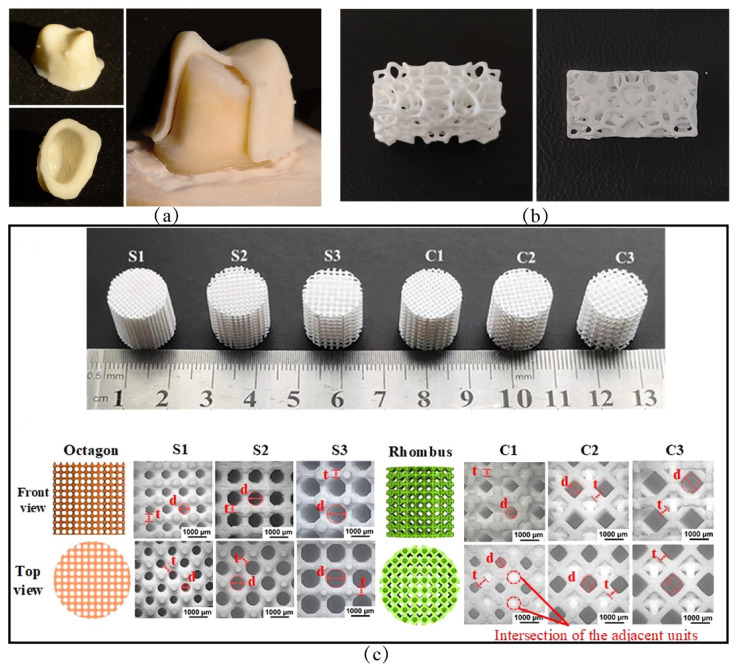
Biomedical applications of VPP-printed Al_2_O_3._: (**a**) photographs of an SLA-manufactured Al_2_O_3_ dental crown framework [172], copyright 2017, with permission from Elsevier; (**b**) bone structures printed by DLP before (**left**) and after (**right**) sintering [173], copyright 2022, with permission from Elsevier; (**c**) octagonal and rhombic bone scaffolds (S is octagonal; C is rhombic) [108], copyright 2022, with permission from Elsevier.

Al_2_O_3_ ceramics are inherently chemically stable, have excellent biocompatibility, and have been widely applied in a variety of biomedical fields, such as dental implants, orthopedic implants or drug release carriers. For traditional subtractive manufacturing, it is difficult to produce biological devices, and due to the complex geometrical structure of biological devices, this results in the wastage of raw materials and increases the manufacturing cost. This provides an opportunity for the biomedical applications of VPP. Al_2_O_3_ ceramics via VPP can not only achieve personalized size and shape control, but also greatly save manufacturing costs and improve manufacturing precision [171]. Figure 10 shows the biomedical applications of VPP-printed Al_2_O_3_ parts_._

Dehurtevent et al. [172] compared the mechanical properties of dental ceramics printed from light-cured Al_2_O_3_ ceramic slurries with different particle sizes and solids contents, and found that, when the solids content reached more than 80%, Al_2_O_3_ crowns comparable to the strength of subtractive manufacturing can be produced. Esteves et al. [173] combined the computer-aided design (CAD) and DLP technique to incorporate hydroxyapatite into Al_2_O_3_ ceramic matrices to enhance their bioactivity and biocompatibility. Then, composite ceramic slurries composed of these two were printed to generate a customized bone structures. Lei et al. [108] designed and fabricated two types of ceramic scaffolds with different porosities via VPP, namely octagonal and rhombic scaffolds. The pore diameters are 0.566 mm and 1.0 mm, respectively, while elastic moduli are correspondingly 3834.9 MPa and 599.8 MPa, which are suitable for bone tissue engineering. All these examples showed that the VPP-printed Al_2_O_3_ ceramics have promising application prospects in the biomedical field.

### 5.5. Large-Size Additive Manufacturing Based on VPP

Large-size fabrication based on VPP is always a difficulty due to the nature of its layer-by-layer curing. In the case of DLP, for example, the increase in the UV projection area results in a lower feature resolution because the DMD of DLP optical machines has a limited number of micromirrors. Additionally, the ceramic slurry does not have a high enough solid content, and the microstructural changes caused by the debinding process are difficult to control for large-size light-curing fabrication. Gu et al. [174] used an advanced top–down DLP technique called Matrix-DLP, which seamlessly connects two high-resolution DLP projectors. The final projector has high resolution and a large projection area. A high solid-loaded Al_2_O_3_ ceramic slurry designed for top–down DLP was developed using a novel surface modifier for Al_2_O_3_ powder. A segmented atmospheric debinding method was also designed. Finally, Al_2_O_3_ ceramic parts with a diameter of 140 mm and a thickness of 12.59 mm were obtained with a good interlayer bonding, high density of 99.4%, and high microhardness of 17.92 GPa.

The VPP printing of large-size Al_2_O_3_ parts is firstly limited by the development of shaping equipment, and the size of current light-curing devices is limited. Moreover, the technology for generating UV light over a large area is still in development and prone to uneven print quality and low precision. Secondly, the large size makes the inherent brittleness of ceramic parts more dangerous, and controlling the structure and properties of large-size parts is inherently more difficult. Therefore, the VPP printing of large-size Al_2_O_3_-based ceramic parts is still in its infancy.

### 5.6. Applications of VPP-Printed Al_2_O_3_ Ceramic Substrates in the Electronics Industry

With the development of the electronics industry, there has been an increasing requirement for microelectronic systems that can work properly in higher-temperature environments, which has high requirements for the performance of electronic substrates. This needs to conform to fixed shapes and have a strong performance at elevated temperatures, and Al_2_O_3_ ceramic substrates produced by VPP exhibit great advantages in this regard. Alhendi et al. [175] developed a microelectronic system that operates at a temperature of 850 °C, in which an Al_2_O_3_ ceramic substrate with a very high purity (99.8%) was produced by VPP. Negi et al. [176] used the VPP method to prepare thin Al_2_O_3_ ceramic substrates with a thickness under 300 µm and control their warpage. Through Fourier transform infrared spectroscopy (FTIR) characterization, the layer-by-layer curing during the VPP process results in the warpage of the molded parts. For a ceramic substrate with the dimensions of 40 × 10 × 0.3 mm, warpage was observed to be 3–5 mm after the heat treatment. A post-curing technique was used to reduce the curing gradient, and finally realize the reduction in warpage by 80%. Additionally, 3D-printed Al_2_O_3_ ceramics are also used as heat sinks with complex structures for electronic systems. Dang et al. [177] prepared Al_2_O_3_ ceramic heat sinks with micro-channels using DLP. With an Al_2_O_3_ ceramic heat sink and a water flow of 50 mL/min, the temperature of the laser diode module is kept below 61 °C, even with a drive power of up to 115.6 W.

However, the dielectric constant and loss angle tangent of VPP-3D printed Al_2_O_3_ substrates are affected by porosity and impurities, which may not meet the low loss requirements for high-frequency substrates. The thermal conductivity of the parts may be lower than that of traditionally molded alumina ceramics due to the limitations of layer printing, which also limits their applications in heat dissipation for the thermal management of high-power devices.

### 5.7. Ceramic Filter Membranes

**Figure 11:**
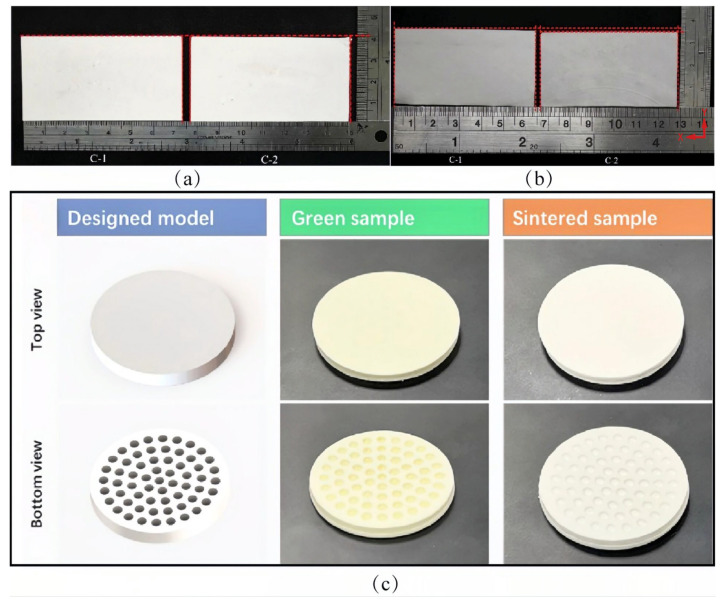
Al_2_O_3_ ceramic filter membranes printed by VPP: (**a**) ultrafiltration membranes obtained by 3D printing [178], copyright 2022, with permission from Elsevier; (**b**) fully densified ultrafiltration membranes [178], copyright 2022, with permission from Elsevier; (**c**) Al_2_O_3_ ceramic membranes fabricated by DLP: designed model (**left**), green body (**middle**), sintered body (**right**) [179], copyright 2022, with permission from Elsevier.

Al_2_O_3_ ceramic filter membranes have more applications in food, drug, and other purification fields due to the high flux, chemical stability, and good anti-fouling properties. However, the preparation of ceramic filter membranes formulated with complex shapes is still challenging. VPP can precisely solve this challenge in the preparation of Al_2_O_3_ ceramic membranes. Figure 11 shows some Al_2_O_3_ ceramic filter membranes by VPP.

For the pore size and number of ceramic Al_2_O_3_ filter membranes, this can be solved by oriented structural design. Moreover, the printing size precision of VPP is good, which can control the relatively uniform size of holes. However, the debinding/sintering shrinkage has a large impact on the shaping accuracy, so the design of a suitable debinding/sintering process to ensure a stable shrinkage is one of the key factors in the preparation of ceramic filter membranes.

Ray et al. [178] used a new method called solvent-based slurry stereo lithography to produce the Al_2_O_3_ ceramic filter membranes for ultrafiltration applications. There exist closed pores with an estimated particles size range of 2–3 μm. Wang et al. [179] used the DLP method to fabricate Al_2_O_3_ ceramic membranes, which have a porosity of 42.6%, an average pore size of 0.9 μm, and a purified water flux of 2.66 m^3^ m^−2^ h^−1^ bar^−1^. Chen et al. [180] introduced TiO_2_ nanoparticles to release the pores occupied by Al_2_O_3_ nanoparticles and enhance the connection between micrometer-sized Al_2_O_3_ particles. In this case, composite ceramic membrane carriers were successfully prepared by printing the TiO_2_-Al_2_O_3_ composite slurry using the LCD method. The optimized membrane has a pore size of 1.36 μm and a porosity of 42.4%. The pure water flux is 3700 m^3^ m^−2^ h^−1^ bar^−1^.

## 6. Conclusions and Prospect

This paper provides a scoping review to introduce the principle of vat photopolymerization (VPP) and summarizes the factors influencing the VPP process of Al_2_O_3_ ceramics including slurry formulation, printing parameters and debinding/sintering processes. In the meantime, the approaches to improving the shaping accuracy and properties of VPP-printed Al_2_O_3_ ceramics are pointed out based on these factors. Researchers can design every aspect of part preparation based on these strategies. The analysis of these factors facilitates a better understanding of the specific effects of VPP technology on shaping accuracy, microstructure, and properties, and helps enhance our understanding of the specific implications of this shift from subtractive to additive manufacturing. Finally, the properties, applications, and challenges of Al_2_O_3_ ceramic parts via VPP in some key fields are listed. In summary, this paper draws the following conclusions:

(1) This review first focuses on the effect of ceramic slurries on the accuracy and property of VPP-printed Al_2_O_3_ parts. The rheological properties and solid contents of the slurries are the most critical properties. The effects of particle size distribution, morphology, particle surface modification, resin, and dispersant on the rheological properties of slurries are discussed to obtain a better understanding on the underlying mechanisms. The selection of moderate particle sizes and regular shaped particles improves the slurry viscosity. By regulating the resin and dispersant system, the rheological properties of the slurry are significantly improved. In addition, the effect of composite components in the slurry on viscosity and curing depth is also considered for improving the comprehensive properties, including structural/functional integration applications.

(2) The structure of Al_2_O_3_ as well as the principal additive resins and dispersants are the key to improving printing properties of Al_2_O_3_. The review describes different crystal structures and the main properties of alumina, and summarizes the commonly used resin systems and dispersants. It is instructive for the composition design of VPP printing slurries.

(3) The shift in thinking from subtractive to additive manufacturing is a turning point in the evolution of manufacturing, which facilitates the personalization and design of structural flexibility and complexity for a variety of products. The structural design and optimization is an important part of VPP molding. The innovation and optimization of Al_2_O_3_ topology through CAD and other 3D modeling software is a major development for VPP-printed Al_2_O_3_. The topology design methods are described regarding a variety of VPP-printed Al_2_O_3_ and structures that have been considered in recent years. Design ideas for porous, lattice, and bionic structures are presented.

(4) The key to VPP molding is to modulate the interaction between ceramic particles and UV light. This paper introduces the photosensitive resin systems commonly used for the photopolymerization of Al_2_O_3_-based slurries, and reveals the regulation strategies of print parameters based on a basic understanding of their effects on layer thickness, curing depth, laser pitch, and other parameters. Specifically, thinner layer thicknesses and a longer exposure time can improve the curing depth, but internal stresses from overcuring may need to be taken into account.

(5) Post-process treatments consist mainly of debinding and sintering. The effects of temperature, heating rate, holding time, and atmosphere on the debinding/sintering process are analyzed. The influences of sintering additives on the grain size and properties of Al_2_O_3_-based composites after sintering are also summarized. The designer can choose suitable sintering aids for purposes such as reducing the shrinkage or lowering the sintering temperature. This paper discusses the mechanisms and control methods for the defects generated during the debinding/sintering process. By adding some porous agents, it is possible to create channels for debinding and thus reduce defects. In fact, exploring the mechanisms of defect generation in debinding/sintering is still in the exploratory stage and becomes an important direction that needs to be developed in the future.

(6) VPP is one of the future directions of precision manufacturing, which provides a new path for the precision molding of Al_2_O_3_ ceramics. VPP can realize the molding of complex structures, which greatly broadens the structural/functional integration applications of Al_2_O_3_ ceramics. However, the preparation of large-sized alumina parts by VPP, the suppression of defects and the porosity generated in debinding/sintering, cracking and stress concentration due to laminated structures all pose challenges to the development of the technology. Nowadays, the alumina ceramics produced by VPP have already made their mark in various fields of ceramic cores, chemical catalysis, friction and lubrication, microelectronic systems, ceramic filter membranes and so on.

## Figures and Tables

**Table 2:** Resins and dispersants used in the photopolymerization of Al_2_O_3_ ceramic slurry.

Al_2_O_3_ D50 (μm)	Resin	Dispersant	Photoinitiator	Solid Content	Viscosity (Pa·s)	Ref.
0.4	MA	PEI + OA	TPO	35 vol.%	10 at 100 s^−1^	[86]
0.3:3 = 3:7	HDDA	MPDISP (4.1 wt.%)	TPO	75 wt.%	1 at 30 s^−1^	[33]
0.5	HDDA	BYK103 (2 wt.%)	BAPO	45 vol.%	0.52 at 30 s^−1^	[88]
0.4	PEGDA	BYK-111	Photoinitiator 819	40 vol.%	0.25 at 30 s^−1^	[89]
0.8	MMA	Tetrahydrofuran (2 wt.%)	Camphor quinone	83 wt.%	-	[90]
1	ACMO	MPDISP (2.23 wt.%)	TPO	72.5 wt.%	1 at 400 s^−1^	[91]
4	HDDA:PUA= 1:1	-	TPO-L	56 vol.%	0.52 at 50 r/min	[92]
4.42	HDDA,ETPTA	-	Camphor quinone	-	-	[93]
0.18	HDDA:EA= 4:1	Disperbyk-111 (2.5 wt.%)	TPO	40 vol.%	0.5 at 10 s^−1^	[94]
0.18	HDDA:PEG400DA = 1:4	Disperbyk-111 (4.5 wt.%)	TPO	40 vol.%	1.5 at 10 s^−1^	[94]
5.1	HDDA,TMPTA, NPG2PODA	BYK-111	Irgacure 651	-	9.171 at 30 r/min	[44]
0.15	TEGDMA: PPGDMA: 2-EH = 1:1:1	KD1 (1.5 wt.%)	Omnirad 819	45 vol.%	1.9 at 10 s^−1^	[95]
1	HDDA:HEMA:TMP3E0TA:PPTTA = 6:2:1:1	KOS110 (5 wt.%)	TPO	50 vol.%	1.1 at 30 s^−1^	[96]

**Table 3:** The rheological properties of Al_2_O_3_ composite slurry.

Al_2_O_3_ D50 (μm)	Additives	Content	Solid Content	Viscosity (Pa·s)	Curing Depth (μm)	Ref.
0.2	TiO_2_ (0.2 μm)	5 wt.%	50 wt.%	0.92 at 100 r/min	-	[97]
0.2	ZrO_2_ (0.2 μm)	20 wt.%	65 wt.%	0.2	-	[98]
0.2	ZrO_2_ (<0.2 μm)	15 vol.%	40 vol.%	-	60	[99]
28.3	Ce-ZrO_2_	-	62.5 wt.%	10 at 10 s^−1^	45	[100]
0.39	Graphene (3–10 nm)	0.07	60 wt.%	25 at 50 s^−1^	65	[101]
0.2	Y_2_O_3_ (0.5 μm)	5 wt.%	79 wt.%	1.97 at 13 s^−1^	83 ± 2.51	[102]

**Table 4:** The effects of debinding and sintering processes on the properties of Al_2_O_3_ via VPP (※ measured from SEM micrographs).

Al_2_O_3_ D50 (μm)	Debinding	Sintering	Density	Flexural Strength (MPa)	Hardness (GPa)	Fracture Toughness (MPa·m^1/2^)	Porosity	Grain Size (μm)	Application	Ref.
60.85, 5.1	1050 °C (N_2_)	2 °C/min 1700 °C, 1.5 h	2.04 g/cm^3^	51.36	-	-	47.93%	40–50	High-resolution porous ceramics	[44]
5.81: 1.14 = 9; 1	0.5 °C/min 600 °C, 2 h (Ar) 2 °C/min 600 °C, 2 h	5 °C/min 1550 °C, 2 h	-	78.15 ± 3.50	-	-	30.12%	2–10 ※	Alumina mold material	[117]
4.42	1000 °C	1600 °C, 4 h	-	19.62	-	-	-	1–10 ※	Functional gradient ceramics	[93]
0.5	1000 °C, 1 h	1600 °C, 5 h	99%	471	17.31	-	-	1–5 ※	Mechanical components	[118]
0.25	-	1 °C/min 1600 °C, 2 h	3.986 g/cm^3^	650	-	3.13	-	4–10 ※	Mechanical components	[119]
0.18	1150 °C, 2 h	1600 °C, 1.5 h	3.86 g/cm^3^	540	-	-	-	0.2–1 ※	-	[85]

**Table 5:** The effects of the debinding and sintering process on the properties of 3D-printed Al_2_O_3_-based composites (※ measured from SEM micrographs).

Materials	Debinding	Sintering	Density	Flexural Strength (MPa)	Hardness	Fracture Toughness (MPa·m^1/2^)	Grain Size (μm)	Application	Ref.
Al_2_O_3_ (0.2 µm) +ZrO_2_ (0.2 µm, 15 vol.%)	600 °C, 1 h	1625 °C, 1 h	4.22 ± 0.03 g/cm^3^	693 ± 87	21.14 ± 1.64 GPa	-	0.75–2 ※	Structural ceramics	[99]
Al_2_O_3_ (0.2 µm) +ZrO_2_ (0.2 µm, 20 wt.%)	1000 °C, 0.5 h	1600 °C, 1 h	4.28 g/cm^3^	-	17.3 GPa	5.1	0.5–2 ※	-	[98]
Al_2_O_3_ (0.2 µm) +ZrO_2_ (0.2 µm, 15 vol.%)	550 °C, 5 h	1500 °C, 1 h	3.75 g/cm^3^	-	14.1 GPa	4.05	1–5 ※	Ceramic gear	[138]
Al_2_O_3_ (0.2 µm) +ZrO_2_ (0.2 µm, 20 wt.%)	1000 °C, 0.5 h	1600 °C, 4 h	4.26 g/cm^3^	530.25	17.76 GPa	5.72	0.25–2.25	-	[139]
Al_2_O_3_ (0.2 µm) +TiO_2_ (0.2 µm, 5 wt.%)	1100 °C, 1 h	1600 °C, 2 h	2.96 g/cm^3^	4.24	-	-	0.2–0.3	Bioceramics	[97]
Al_2_O_3_ (0.2 µm) +CaO (0.2 µm, 5 wt.%)	550 °C, 2 h	1650 °C, 2 h	1.8 g/cm^3^	24	-	-	2–10 ※	Ceramic core	[140]
Al_2_O_3_ (0.39 µm) +Graphene (0.07 wt.%)	-	1650 °C, 1.5 h (vacuum)	99.7%	-	18.61 GPa	-	4–20 ※	-	[101]
Al_2_O_3_ (0.39 µm) +C_f_ (0.2 wt.%)	1550 °C, 2 h	700 °C	3.2 g/cm^3^	15	-	-	1–5 ※	-	[103]
ZTA (20 wt.%ZrO_2_)+MnO (0.5 wt.%)	360 °C, 120 h (N_2_)	1600 °C, 2 h	4.0 g/cm^3^	-	1409 ± 50 HV	-	0.8–4 ※	Bioceramics	[141]

**Table 6:** The effect of sintering additives on properties of 3D-printed alumina ceramic parts.

Sintering Aid	Effect	Density	Flexural Strength (MPa)	Shrinkage X Axis	Shrinkage Y Axis	Shrinkage Z Axis	Refs.
TiO_2_ (1.5 wt.%)	Enhance strength	3.58 g/cm^3^	216.7	15%	14%	13.5%	[142,143]
CaCO_3_ (1.5 wt.%)	Reduce shrinkage and strength	2.95 g/cm^3^	105.8	10.5%	9.5%	7%	[142]
MgO (1 wt.%)	Enhance density and strength	2.99 g/cm^3^	111.1	10.8%	10.7%	9.6%	[143,144,146]
Y_2_O_3_ (1 wt.%)+ MgO (0.5 wt.%)	Enhance density and strength	96.75%	491.6	21.4%	21.66%	26.05%	[144]
CuO-Mg(OH)_2_-TiO_2_ (3.5 wt.%)	Reduce sintering temperature	2.77 g/cm^3^	301	19.2%	19.1%	19.5%	[145]

## Data Availability

No new data were created or analyzed in this study.
